# Molecular Regulation of Muscle Satellite Cell Self-Renewal

**DOI:** 10.4172/2157-7633.S11-e002

**Published:** 2012-11

**Authors:** Norio Motohashi, Atsushi Asakura

**Affiliations:** Stem Cell Institute, Paul and Sheila Wellstone Muscular Dystrophy Center, Department of Neurology, University of Minnesota Medical School, MN, USA

Skeletal muscle possesses a remarkable ability for regeneration and can go through rapid repair following muscle injury. This regeneration depends on the activity and contributions of muscle satellite cells, which are located between the sarcolemma of myofibers and the basal lamina [1]. Upon muscle injury, satellite cells are activated, driven out of their quiescent state, and start to proliferate. Proliferating satellite cells, termed myogenic precursor cells, or myoblasts, then exit cell proliferation, differentiate into myocytes, and fuse with either each other or with existing myofibers in order to repair injured muscle [2].

During myogenesis, these satellite cells can be distinguished based on Pax7 and MyoD expression; both are essential myogenic transcription factors. Pax7+MyoD− cells are in a quiescent state, Pax7+MyoD+ cells are proliferating, and Pax7−MyoD+ cells are undergoing differentiation [3,4]. In addition to cell proliferation and myofiber differentiation, activated satellite cells also have an important function in maintaining their own satellite cell reserve pool through self-renewal, which is essential for continuous muscle regeneration throughout the life of an animal. Freshly isolated satellite cells and activated satellite cells can contribute to both regenerating myofibers and quiescent satellite pool after transplantation, strongly indicating that satellite cells possess the bipotentiality that contributes to both myogenic regeneration and replenishment of satellite cell pool [5,6]. Since the myogenic differentiation potential and the number of satellite cells decline as muscle ages, the regeneration capacity of muscle is also decreased in aging. It also has been reported that loss of myogenic differentiation capacity of satellite cells due to the continual need for regeneration may contribute to disease progression in muscular dystrophy [7,8]. Genetic ablation experiments using Pax7-null mice, whose satellite cell numbers are very low, exhibited a defect where the remaining satellite cell population proliferated and differentiated in vitro along with the failure of muscle growth and regeneration in vivo [9,10]. Thus, the maintenance of satellite cells is necessary for continuous muscle regeneration, especially in aging muscle. Although accumulating evidences characterize the molecular mechanisms of myogenesis, the regulation of how satellite cells maintain their numbers or functions is not completely understood.

Currently, there are two mechanisms that attempt to define the maintenance of the muscle satellite cell pool in adult muscle. The first mechanism suggests that satellite cells are a functionally heterogeneous population while the other states that satellite cells are capable of self-renewal to generate a stem cell pool after cell division. Previous studies have proposed that satellite cells are composed of two different populations: One population consists of myogenic satellite cells, which possess myogenic differentiation potential. The other population is an undifferentiated subgroup which retains the satellite stem cell profile [5,11,12]. This idea is supported by a recent study in which satellite cells could be distinguished as fast- and slow-dividing cells through labeling with fluorescent lipophilic dye (PKH26) [13]. Fast dividing cells gave rise to a higher number of myogenic differentiated cells while slow dividing cells represented quiescent-like self-renewing cells. Transplantation experiments demonstrate that slow dividing cells possess stem cell like potential, and contributed to continuous muscle regeneration in vivo, suggesting that slow dividing cells are able to produce myogenic stem cell progeny. In this study, gene expression analysis in slow dividing cells demonstrated some important clues in distinguishing the “stem cell” population from the myogenic population. Developments of this nature address progress toward more specific means of identifying and isolating “stem cell” population satellite cells such as use of endogenous cell surface markers using FACS. Further studies will be expected to clarify these approaches.

A small population of satellite cells, possessing a potential of “stemness”, is capable of maintenance of the number of satellite cells as proved by transplantation experiments. This small cell niche also produces myoblasts during muscle regeneration [13], indicating that “self-renewal” of satellite cells should be important to maintain the quiescent satellite cell pool. Several papers have demonstrated that regulation of Pax7 and MyoD expression in satellite cells is crucial for satellite cell self-renewal and myogenic differentiation processes. During myogenic differentiation, the majority of Pax7+MyoD+ myoblasts undergo MyoD+ differentiation into myocytes and myotubes with attenuation of Pax7. By contrast, a small number of myoblasts maintain Pax7 expression while decreasing MyoD expression and undergo satellite cell self-renewal to a quiescent state to resume a place in the stem cell niche. These observations suggest that most satellite cells may determine their cell fates after cell proliferation and that the down-regulation of MyoD expression could be an important factor for the process of satellite cell self-renewal.

MyoD belongs to the myogenic basic helix-loop-helix (bHLH) transcription factors that play essential roles in myogenic specification, differentiation and maintenance during muscle development and regeneration [14]. MyoD specifically serves as a potent myogenic master transcription factor that can reprogram many non-muscle cell types to a myogenic lineage when expressed in those cells [15]. Recently, Asakura et al. [16] reported that following myoblast injection into regenerating mouse muscle, engraftment and survival of myoblasts isolated from adult MyoD−/− mice was significantly increased compared to wild-type myoblasts. In addition, transplanted MyoD−/− myoblasts, but not wild-type myoblasts, could give rise to the satellite cell compartment in muscle. Importantly, the MyoD−/− myoblasts were much more resistant to apoptosis compared to wild-type myoblasts [17]. In addition, MyoD−/− muscle contains an increased number of satellite cells [17–19]. Therefore, MyoD−/− wild-type myoblasts preserve stem cell characteristics including resistance to apoptosis, efficient engraftment and contribution to the satellite cell compartment following transplantation. Taken together, these observations strongly suggested that decline of MyoD expression is a key factor for myoblast to satellite cell self-renewal process.

Six1, which belongs to Six homeoprotein family and is expressed in quiescent and activated satellite cells, is reported to regulate skeletal muscle regeneration as well as satellite cell self-renewal [20]. Expression of Six1 can activate MyoD and myogenin expression followed by myogenic differentiation. By contrast, the absence of Six1 disturbs muscle regeneration and increases the number of satellite cells. Down-regulation of Six1 and Dusp6, which are inhibitors of ERK1/2 signaling, induces satellite cell self-renew by the activation of ERK1/2 signaling associating with MyoD suppression. Recent evidence touches on the asymmetric division model of satellite cells during the self-renewal process, which was found to be regulated via the p38α/β MAPK pathway [21]. During myoblast proliferation, asymmetrically localized Par complexes activate p38α/β MAPK signaling, resulting in an increase of MyoD and promotion of myogenic differentiation. By contrast, lack of p38α/β MAPK signaling in myoblasts suppresses MyoD expression, resulting in induction of satellite cell self-renewal. Another study has also explained the regulation of myoblast self-renewal through microRNAs. MiR-489, which is shown to be highly expressed in quiescent satellite cells, functions as the regulator of the satellite cell fate that leads back to the quiescent state by suppressing the expression of the oncogene Dek [22]. Dek expression is activated in proliferating myoblasts expressing MyoD, while Dek is absent in quiescent satellite cells. The downregulation of miR-489 induces the activation of satellite cells through the up-regulation of Dek expression, suggesting that the miR-489-Dek pathway is important for maintenance of quiescent satellite cells. As described above, the factors that downregulate MyoD and up-regulate Pax7 could be crucial to guide satellite cells from the activate state to quiescent state. However, it remains to be elucidated what factors selectively induce asymmetric or symmetric division from the myoblast state.

While several transcription factors are expected to be key elements in sustaining the satellite cell pool, the position of satellite cells in contact with muscle fibers is also expected to hold a comparable level of importance in management of the satellite stem cell niche. A recent paper demonstrates that increased levels of FGF2, typically a stimulator of satellite cell activation, induces loss of satellite cells through decreased Spry1, the downstream target of FGF2, in aged muscle fiber. These results indicate that age-related changes of FGF signaling in the stem cell niche influence altered stem cell maintenance and function [23]. In addition, the Notch signaling pathway is also known as an important contributor to a host of cell functions and has crucial roles in cell-cell commitment and cell fate determination during tissue homeostasis and regeneration [24]. The absence of Notch signaling in satellite cells of adult mice induces spontaneous activation from the quiescent state to rapid differentiation, resulting in depletion of satellite cell pool and the failure of muscle regeneration [25,26]. In addition, the inhibition of Notch target genes such as Hesr1 and Hesr3 also leads to the reduction of satellite cell self-renewal, depleting the satellite cell pool and the consequent impairment of muscle regeneration [27]. Thus, activation of FGF and Notch signalings caused by cell-cell or cell-fiber contact may influence satellite cell proliferation or homing capacity.

In relation to Notch signaling, a recent paper demonstrates interesting results in which the depletion of the satellite cell pool in RBP-J mutant mice, a key mediator of Notch signaling,, is recovered by the deletion of MyoD [28]. In this study, Notch signal mutated coRbpj; MyoD−/− satellite cells were not located in normal position. Instead, they were positioned in the interstitial space of muscle fiber and did not contribute to myofiber growth. Interestingly, Notch signaling is required to produce numbers of basement membrane protein and adhesion molecules such as Integrin-α7, Collagen XVIIIa1, Megf10 and M-CAM, indicating that Notch signaling contributes to homing of satellite cells by stimulating the production of basal lamina.

In addition to cell commitment, recent reports indicate that physiological conditions in satellite cells may influence the regulation of self-renewal. Liu and colleagues have proposed that hypoxia does not affect myoblast proliferation but instead promotes satellite cell self-renewal by up-regulating Pax7 [29]. In fact, increased Notch signaling caused by hypoxia down-regulates miRNA-1/miRNA-206 expression, which targets Pax7. Therefore, decreasing of miRNA-1/206 induces Pax7 expression. Given that MyoD is repressed by hypoxia [30] or Notch signaling [31] as previously reported, the hypoxia condition mediating Notch signaling and microRNAs could be an important factor in regulation of MyoD and Pax7 for satellite cell maintenance.

Thus, accumulating observations demonstrate that maintenance of muscle satellite cells is intricately involved in several biological and physiological processes. Although the idea in which satellite cells are essential for muscle regeneration is widely accepted, the mechanism of cell fate determination, whether satellite cells differentiate into myocytes or maintain as stem cells, is still controversial. In addition, it is of great interest to the field to define the number of satellite cells in adult and aged muscles in addition to what distinct niches regulate satellite cell number and heterogeneity. In order to reveal these mysteries, further exploration that approaches the identity of fundamental mechanisms in satellite cells must be conducted. Recent studies report that satellite cells are divided predominantly by asymmetric template DNA segregation, generating daughter cells which retain stem cell potential and myogenic committed cells [32]. In addition, histone methylation by methyltransferase complexes recruited by methylated Pax7 play an important role in the epigenetic regulation of Myf5 as a Pax7 downstream gene and the determination of stem cell fate [33]. Therefore, these phenomena represented in satellite cells are inherent mechanisms involved in DNA methylation pattern or epigenetic memory, and future studies may provide us new insights of the regulation for satellite cell differentiation, self-renewal and maintenance.
